# MAPK signaling pathway-based glioma subtypes, machine-learning risk model, and key hub proteins identification

**DOI:** 10.1038/s41598-023-45774-0

**Published:** 2023-11-04

**Authors:** Hengrui Liu, Tao Tang

**Affiliations:** 1 Xinkaiyuan Pharmaceuticals, Beijing, China; 2 Guangzhou Regenerative Medicine Research Center, Future Homo Sapiens Institute of Regenerative Medicine Co., Ltd (FHIR), Guangzhou, China; 3 Department of Molecular Diagnostics, Sun Yat-Sen University Cancer Center, State Key Laboratory of Oncology in South China, Guangzhou, China; 4 Collaborative Innovation Center for Cancer Medicine, Guangzhou, China

**Keywords:** Cancer, Cancer genetics, Cancer prevention, Oncogenes

## Abstract

An early diagnosis and precise prognosis are critical for the treatment of glioma. The mitogen‑activated protein kinase (MAPK) signaling pathway potentially affects glioma, but the exploration of the clinical values of the pathway remains lacking. We accessed data from TCGA, GTEx, CGGA, etc. Up-regulated MAPK signaling pathway genes in glioma were identified and used to cluster the glioma subtypes using consensus clustering. The subtype differences in survival, cancer stemness, and the immune microenvironment were analyzed. A prognostic model was trained with the identified genes using the LASSO method and was validated with three external cohorts. The correlations between the risk model and cancer-associated signatures in cancer were analyzed. Key hub genes of the gene set were identified by hub gene analysis and survival analysis. 47% of the MAPK signaling pathway genes were overexpressed in glioma. Subtypes based on these genes were distinguished in survival, cancer stemness, and the immune microenvironment. A risk model was calculated with high confidence in the prediction of overall survival and was correlated with multiple cancer-associated signatures. 12 hub genes were identified and 8 of them were associated with survival. The MAPK signaling pathway was overexpressed in glioma with prognostic value.

## Introduction

Last year, 308,102 new cases of brain cancer were diagnosed resulting in 251,329 cancer-related deaths globally^1^. As the most prevalent malignant tumor disease in the brain, glioma represents the major type of brain tumor and its incidence has been increasing in the past several years^2,3^. Glioblastoma is a type of brain cancer with a low survival rate. Over a five-year period, only about 6.8% of glioblastoma patients survive. On average, these patients live for about 8 months^2,3^.

Clinical gliomas are graded into WHO grades 1–4. The Cancer Genome Atlas program classified grade 2–3 glioma as "low-grade glioma" due to their similar molecular and genetic characteristics. In the past, tumor grades in the central nervous system (CNS) were designated using Roman numerals. However, the fifth edition of the World Health Organization's Blue Books now uses Arabic numerals for grading tumors. The general grading of CNS tumors was abandoned and grading within each tumor entity (histologic subtypes such as astrocytoma, glioblastoma, oligodendroglioma, and oligoastrocytoma) was adopted, which indicates a wide acceptance that the tumor entity rather than the overall grade is more important for patient survival^4^. In TCGA, glioblastoma was separated from the other glioma data cohort because of its genetic difference from the other glioma entity, and it is called “GBM” referred to as “glioblastoma multiforme (GBM)”. In this study, we analyzed data on both low-grade glioma (LGG) and glioblastoma (GBM) in order to investigate the MAPK pathway in glioma as a whole.

Exams for preoperative diagnostics of glioma include brain MRI, biopsy, and genetic detection in the plasma and cerebrospinal fluid^5^. The clinical prognosis of glioma is based on the histologic subtypes of gliomas, genetic subtypes, the stage, and the location of the tumor^6^. The standard treatment for glioblastoma (GBM) typically involves surgery followed by a combination of fractionated external beam radiotherapy and chemotherapy for six and a half weeks. This is followed by a six-month regimen of chemotherapy administered once a month for five days. There is currently no established standard of care for recurrent or progressive GBM, and treatment options may include supportive care, surgery, re-irradiation, systemic therapies, and combined modality therapy^7^. However, so far, glioma is still one of the most deadly diseases among all cancer types and the patient has an extremely poor prognosis^8^. For clinical glioma treatment, early diagnosis and precise prognosis are critical for the early intervention of the disease and the prevention of developing a higher-grade glioma. While several biomarkers have been identified as useful for predicting the prognosis of glioma, there is a need to develop additional biomarkers and prognostic strategies in order to improve our understanding of the overall prognosis of glioma.

One of the signaling pathways that were suggested to potentially be involved in glioma was the mitogen‑activated protein kinase (MAPK) signaling pathway^9,10^. The MAPK signaling pathway is a well-researched pathway that plays a role in many different biological processes within cells. It consists of a series of three kinases: MAPKKK, MAPKK, and MAPK. These kinases act in a cascading fashion. The third kinase MAPK reacted with a wide variety of cellular substrates that function in cells^11^. The MAPK signaling pathway interacts with many bioactive regulators within cells that are involved in cancer, such as hormones, cytokines, and growth factors, as well as internal stress signals and external signals from the environment^12^. Therefore, the expressions of MAPK signaling pathway genes are associated with cancer development.

In cancer, MAPK has been suggested to impact drug resistance and sensitivity^13^. Studies have developed target therapy targeting MAPK signaling for tumor treatments^14,15^. Drugs targeting this pathway have been studied in the glioma field^16–18^. According to many in vitro studies, the MAPK pathway is a downstream target of various cancer-associated regulators and plays a role in mediating the effect of these regulators on glioma cells^19–21^. Previous research on glioma has shown that many cases of pediatric low-grade glioma have genetic mutations and changes that lead to the activation of the MAPK pathway. These genetic changes included BRAF mutation V600E, BRAF fusion, and NF1 mutation^22^. However, to date, most of these studies were limited to low-grade glioma diagnosis, and the study exploring the prognostic values of the MAPK signaling pathway remains lacking. Moreover, recently developed bioinformatic methodologies and databases have not been applied in the study of this pathway in glioma.

This study aimed to study the effects of the MAPK signaling pathway on overall glioma patients and developed a comprehensive risk model for all glioma patients based on the MAPK signaling pathway. To facilitate the application of the MAPK signaling pathway in target medicines, we also conducted computational drug prediction for the key target protein in this pathway. We hope the study can be conducive to future studies in this field.

## Methods

### The acquisition of mRNA sequencing and mutation data

The data with clinical information were obtained from the Chinese Glioma Genome Atlas (CGGA, http://www.cgga.org.cn/index.jsp)^23^, Genotype-Tissue Expression (GTEx, https://gtexportal.org/home/)^24^, and The Cancer Genome Atlas (TCGA, https://www.cancer.gov/ccg/research/genome-sequencing/tcga)^25^, in March 2023. The inclusion and exclusion criteria are provided by TCGA database. LGG and GBM data from TCGA database were downloaded with clinical phenotype information. The three cohorts of CGGA data were downloaded with clinical phenotype information.

### Differential expression gene (DEG) analysis

DEG analysis was conducted using the “limma” package with R. The “ggplot2” package and “pheatmap” package were utilized to plot the figures. Benjamini-Hochberg (BH) Procedure was used to conduct the false discovery rate correction. |log2(Fold change)|> 1 with corrected false discovery rate of < 0.05 was considered as DEG.

### KEGG pathway mapping

KEGG pathway mapping was conducted using the KEGG mapper (https://www.genome.jp/kegg/kegg3a.html). Up-regulated genes in glioma were set in red. Down-regulated genes in glioma were set in yellow. No change genes in glioma were set in green. Data and color setting were sorted out in a .txt file and uploaded to the KEGG mapper for analysis.

### Consensus clustering analysis

The ConsensusClusterPlus^26^ package was used for consistency analysis and clustered the subtypes using R and R studio software. The initial number of clusters was set at 2–6 to obtain the best clustering number. Based on the consensus cumulative distribution function (CDF) plotting out pu from the initial analysis, we select the cluster number when the delta area decreases remarkably. By the NMF method, which is an effective dimension reduction method for cancer subtype identification, patients were clustered into distinct subtypes. The PCA plotting of the subtypes was used to show the different subtypes.

### Cancer stemness

The stemness was accessed as a previous study described^27^. In this method, a one-class logistic regression machine learning algorithm (OCLR) was used to extract transcriptomic and epigenetic feature sets derived from non-transformed pluripotent stem cells and their differentiated progeny. We used the OCLR to calculate the mRNA expression-based stemness index (mRNAsi).

### A machine learning risk model

The prognostic risk model was constructed with the R package “glmnet”^28^ using R and R studio software. The least absolute shrinkage and selection operator (LASSO) regression algorithm (tenfold cross-validation) was utilized to identify gene signatures (selected genes and corresponding coefficient) included in the model. LASSO is a regression analysis method that performs both variable selection and regularization to improve the prediction accuracy and interpretability of the statistical model. This study used LASSO to select the gene signatures and established a model for overall survival prognosis. TCGA data of (LGG and GBM) were used to train the overall survival prognostic model and three cohorts of CGGA data were used to validate the overall survival prognostic model.

### Death risk

The death association was evaluated using the univariate and multivariate Cox analysis^29^. R pack survival [3.3.1] and rms [6.3-0] were used to conduct these analyses with R and R studio software. The analysis was based on overall survival data with TCGA (LGG and GBM) data. TP53 mutation, IDH1 mutation, and histological subtype were included. Factors of P < 0.1 in the univariate Cox analysis were included in the multivariate Cox analysis. Factors of P < 0.05 in the multivariate Cox analysis were regarded as significant. The hazard ratio and p-value were calculated accordingly based on the Cox analysis calculation. Kaplan–Meier (KM) plots^30^ were used to display the survival curve of representative genes. The best p-value cutoff was adopted to separate the samples and conduct the Cox analysis for the KM plot.

### Protein–protein interaction (PPI) network and hub genes

The PPI network was constructed in the STRING tool (https://string-db.org/)^31^ with an interaction score of > 0.9. Active interaction sources included Neighborhood, Co‑occurrence, Databases, Textmining, Experiments, Co‑expression, and Gene Fusion. The hub genes were calculated with “MCC”, “MNC”, “EPC”, and “degree” algorithms using the Hubba (https://apps.cytoscape.org/apps/cytohubba)^32^ in Cytoscape tool (https://cytoscape.org/)^33^.

### Immune analysis

The level of immune cell infiltration was determined using the Xcell algorithms (https://comphealth.ucsf.edu/app/xcell)^34^. With the Tumor Immune Dysfunction and Exclusion (TIDE) algorithm^35^, we also predicted the immune checkpoint blockade (ICB) responses of subtypes. These analyses were conducted using the TIDE online tool (http://tide.dfci.harvard.edu/login/).

### Tumor scores

Pathway scores were calculated for analysis as described^36^. Gene sets were obtained from a previous paper^37^ for these pathways. The ssGSEA algorithm was used to calculate the score for these pathways for each sample. R software GSVA package was used to analyze, choosing parameter as method = 'ssgsea'. The correlation between genes and pathway scores was analyzed by Spearman correlation. A p-value < 0.05 was considered statistically significant. The mRNAsi for the evaluation of stemness and mutation levels of samples were evaluated with the OCLR algorithm^27^ and the tumor mutational burden (TMB) respectively. The TMB was calculated as previously described^38^.

### Drug sensitive

The area under the dose–response curve (AUC) values for drugs and gene expression of a representative gene in cancer cell lines was determined using the GSCALite (http://bioinfo.life.hust.edu.cn/web/GSCALite/)^39^. We integrated drug sensitivity and gene expression data of cancer cell lines in the GDSC^40^ and the CTRP^41^ to conduct the analysis. Spearman correlation analysis was used to calculate the association between gene and drug sensitivity.

### Protein expression analysis

Protein expression levels were accessed and plotted with the UALCAN (https://ualcan.path.uab.edu/index.html)^42^. Representative images of the immunohistochemistry staining of tissues were accessed from the Human Protein Atlas (HPA, https://www.proteinatlas.org/)^43^. Antibody CAB008371 was used in the staining.

## Results

### MAPK signaling pathway genes were overexpressed in glioma

**Figure 1:**
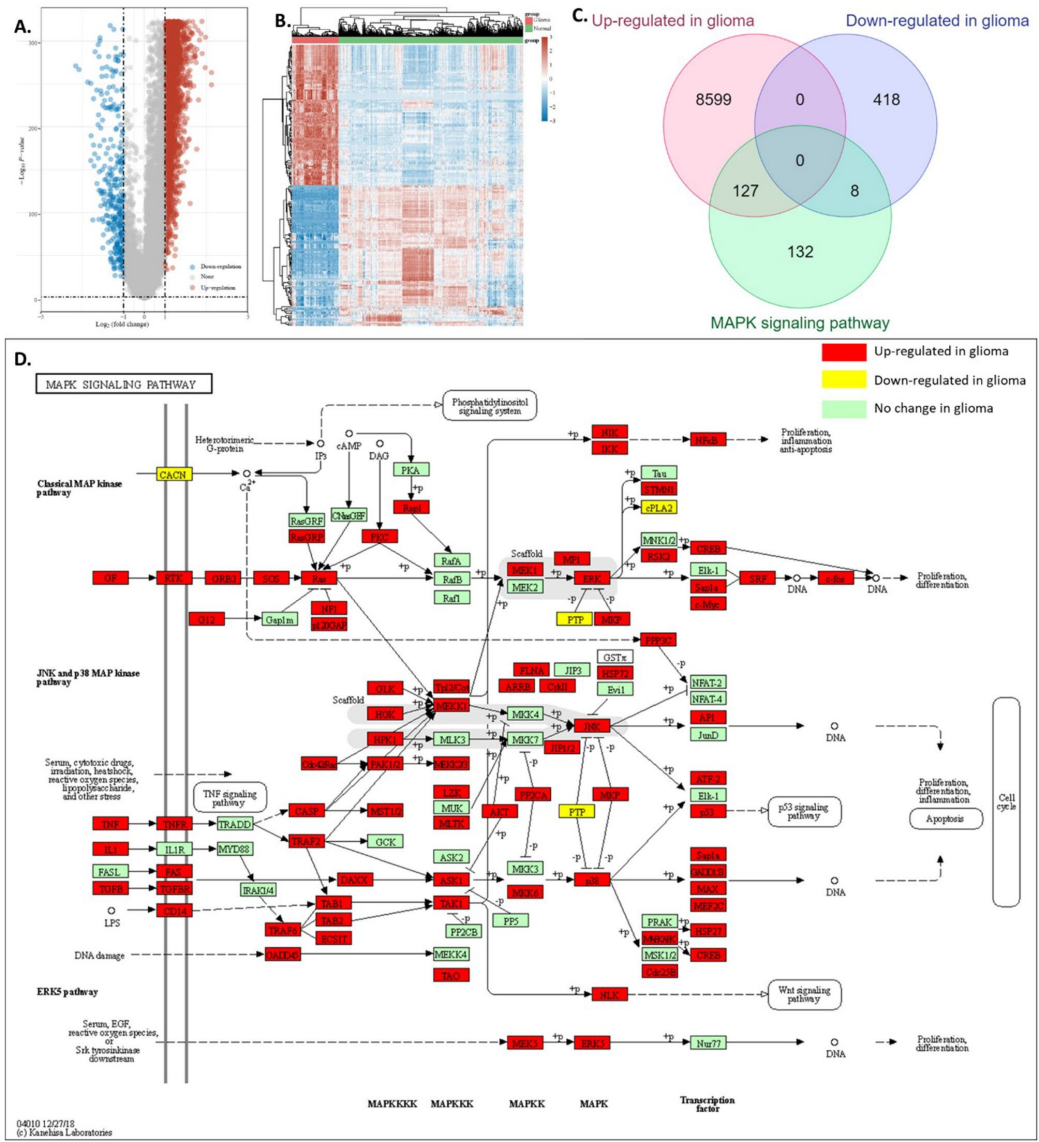
Up-regulations of MAPK signaling pathway genes in glioma. (**A**) Volcano plot of the DEGs between tumor and normal tissues. TCGA and GTEx data were analyzed. (**B**) Heat map showing the identified differential expression genes. (**C**) The intersection analysis of identified DEG and MAPK signaling pathway gene set. (**D**) Mapping of DEG to this pathway.

Firstly, we identified 8726 up-regulated genes and 426 down-regulated genes in glioma compared with normal brain tissues using TCGA and GTEx data (Fig. 1A,B). We also accessed the KEGG MAPK signaling pathway gene set and conducted an intersection analysis with the differential expressed genes in glioma. Results showed that, among 267 MAPK signaling pathway genes, 127 genes in the MAPK signaling pathway gene set were up-regulated in glioma and 8 genes in the MAPK signaling pathway gene set were down-regulated, while 132 genes in the MAPK signaling pathway gene set had no significant differences between tumor and normal tissues (Fig. 1C). The incident of MAPK gene set up-regulated in glioma was 47.6% (127/267), while the incident of MAPK gene set down-regulated in glioma was 3.0% (8/267), the incident of MAPK gene set not altered in glioma was 49.4% (132/267). Detailed expression results of the differentially expressed MAPK genes were provided in S-Fig. 1. We then mapped the regulated genes in glioma to the KEGG MAPK signaling pathway. As shown in Fig. 1D, nearly half of the genes in this pathway were higher (red) with a few lower (yellow) in glioma compared with the normal brain tissues. Therefore, we believed this pathway was a gene signature for glioma and might be useful for clinical applications.

### Glioma cancer subtypes clustering

**Figure 2:**
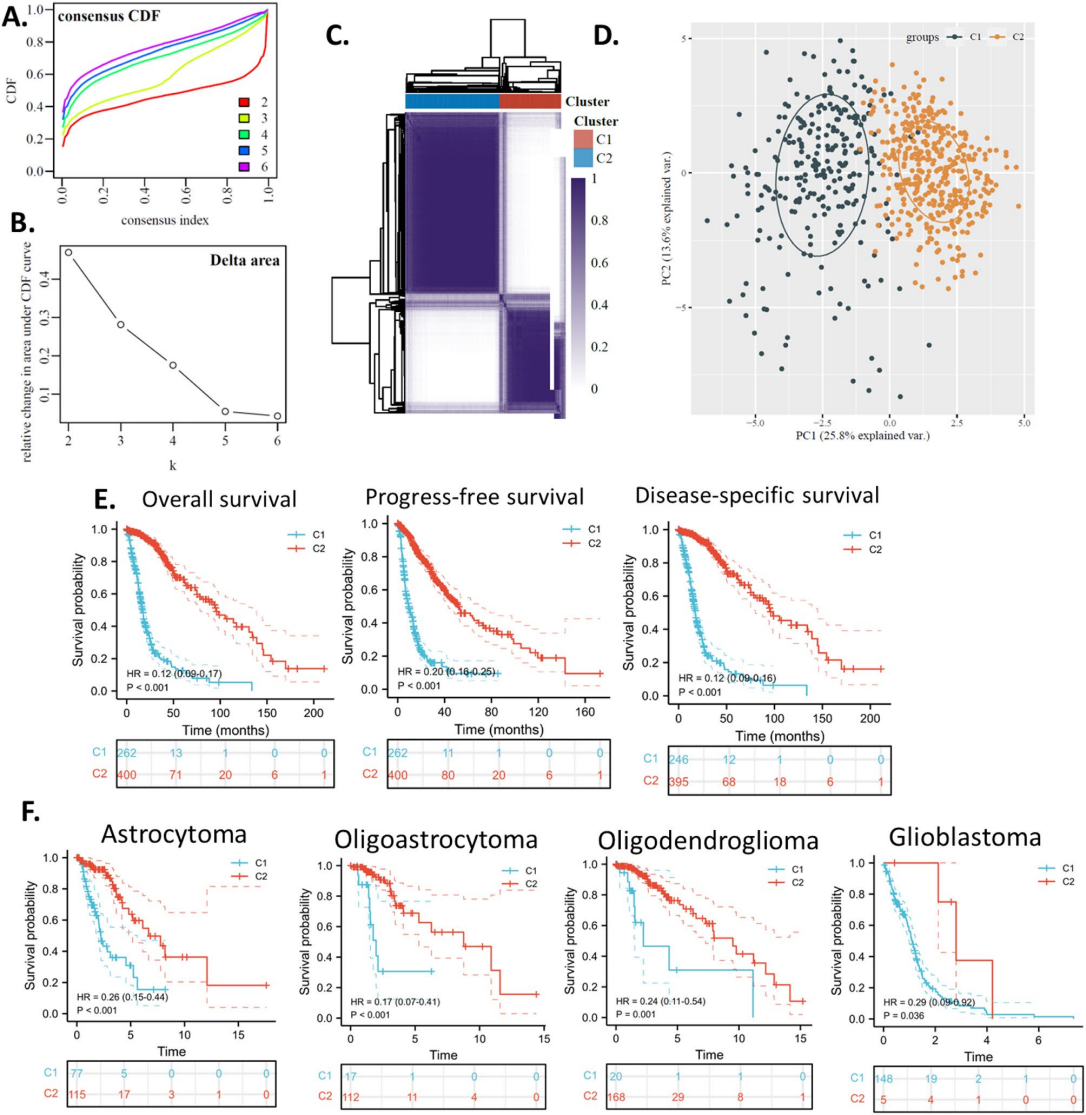
Glioma subtype based on the glioma up-regulated MAPK signaling pathway gene set. The glioma up-regulated MAPK signaling pathway gene set was used to cluster C1/C2 and assign a prognosis. TCGA data were used to calculate all analyses in this figure. (**A**) Consensus CDF plot of subtype numbers (k = 2–6). (**B**) Delta area plot of the consensus CDF plot. (**C**) Cluster trees and consensus matrix of subtypes. (**D**) PCA plot of the consensus clustering. (**E**) survival KM plots of the subtypes. (**F**) Overall survival KM plot of the MAPK subtypes within histologic subtypes. The “ggplot2” package was utilized to plot the figures with R software.

To investigate the MAPK signaling pathway gene signature in glioma, we clustered the glioma sample based on the up-regulated MAPK signaling pathway genes because these genes might be glioma-specific and the overexpression in glioma enables their easy detection. Based on the consensus cumulative distribution function (CDF) plotting, when the number of clusters (K) was 3, the delta area decreased remarkably, thus K = 2 was the optimum cluster number (Fig. 2A,B). By the NMF method, which is an effective dimension reduction method for cancer subtype identification, patients were clustered into two distinct subtypes, which we defined as C1 and C2 (Fig. 2C). The PCA plotting of the subtypes also shows the difference between C1 and C2 (Fig. 2D).

### Glima subtype survival analysis

The most critical difference we found was that the C1 subtype had a significantly worse overall survival, progression-free survival, and disease-specific survival compared to that of the C2 subtype (Fig. 2E). These results indicated that the MAPK signaling pathway gene signature might associate with the survival of glioma patients and can be applied for clinical prognosis. To demonstrate whether the prognostic value of the MAPK subtypes clustered by us was inferior to the already established glioma classification, the widely accepted glioma histologic and genetic subtypes (astrocytoma, glioblastoma, oligodendroglioma, and oligoastrocytoma), we plot the KM curve (Fig. 2F) and conducted survival Cox regression analysis (Table 1) for overall survival of the two MAPK subtypes. First of all, the results showed that the distribution of the C1 and C2 in glioma subtypes was not even. In oligodendroglioma, most of the patients were in C1, while in glioblastoma, most of the patients were in C2. Glioblastoma is high-grade glioma, astrocytoma can have grades 1–4, and oligodendroglioma is grade 2–3 glioma, while oligoastrocytoma is a mixture of astrocytoma and oligodendroglioma^4^. The significant difference between the MAKP subtypes results partly from the uneven distribution of patients in glioma subtypes. Yet, the two MAPK subtypes still showed significant differences within each glioma subtype, suggesting that the MAPK subtypes can provide additional prognostic power to the current glioma subtypes. Cox analysis confirmed that that the MAPK subtypes can provide additional prognostic power to the current biomarkers.

**Table 1:** Cox analysis.

Characteristics	Total (N)	Univariate analysis		Multivariate analysis	
		Hazard ratio (95% CI)	*P* value	Hazard ratio (95% CI)	*P* value
Histological_type	1129				
Astrocytoma	197	Reference		Reference	
Glioblastoma	601	5.146 (3.915–6.764)	**< 0.001**	2.559 (1.763–3.712)	**< 0.001**
Oligoastrocytoma	134	0.632 (0.400–0.996)	**0.048**	0.893 (0.546–1.459)	0.651
Oligodendroglioma	197	0.597 (0.405–0.881)	**0.009**	0.622 (0.392–0.987)	**0.044**
IDH1 mutation	896				
Mutant type	411	Reference		Reference	
Wild type	485	7.740 (6.037–9.924)	**< 0.001**	2.398 (1.592–3.612)	**< 0.001**
TP53 mutation	896				
Mutant type	349	Reference		Reference	
Wild type	547	1.906 (1.551–2.342)	**< 0.001**	1.277 (0.942–1.732)	0.115
Cluster	662				
C2	400	Reference		Reference	
C1	262	8.038 (6.032–10.711)	**< 0.001**	2.454 (1.567–3.843)	**< 0.001**

Significant values are in bold.

### Glima subtype differences

**Figure 3:**
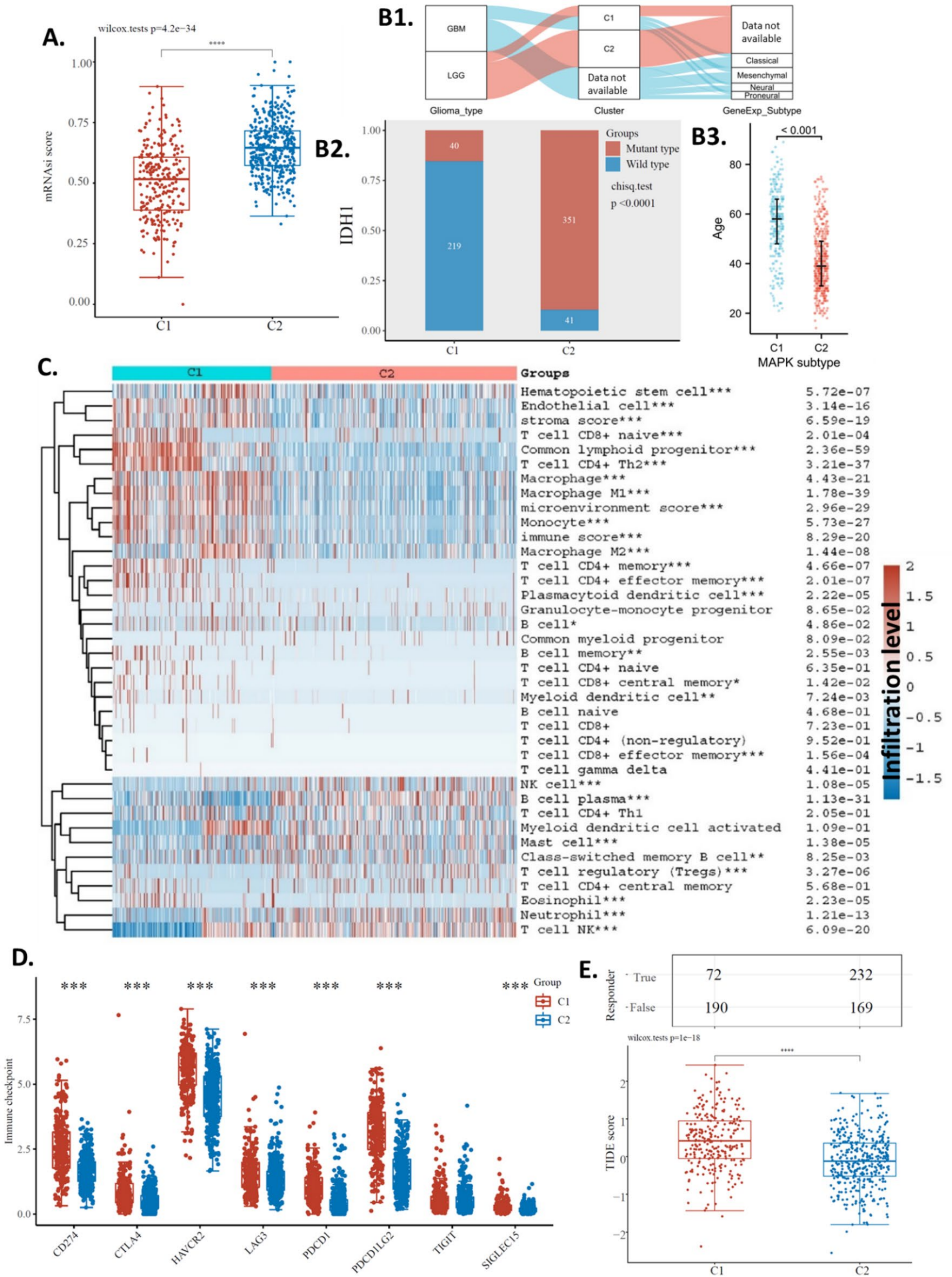
Glima subtype differences. (**A**) Stemness of the subtypes. (**B1**) Comparison Sankey plot of the subtype and current classification of glioma subtyping system. (**B2**) IDH1 mutation rate of the subtypes. (**B3**) Age of the subtypes. (**C**) Heat map of immune cell infiltration of the subtypes. (**D**) Immune check-point levels of the subtypes. (**E**) Predicted ICB response of the subtypes. The “ggplot2” package was utilized to plot the figures with R software.

In addition, we found that the C1 subtype and the C2 subtype were significantly different in cancer stemness (Fig. 3A). To demonstrate the clinical significance of the MAPK subtyping system, this study aimed to clarify tumor subset status with regard to currently well-established categories and check whether the MAPK subtypes match (or not) with them. In this study, we compared our subtypes with LGG/GBM and transcriptomic subtypes: Classical, Mesenchymal, Neural, and Proneural. The Sankey plot showed that all GBM patients fell into C1, while LGG patients fell into C1 and C2 (with more in C2). For the transcriptomic subtypes, C1 patients were distributed relatively evenly among the four transcriptomic subtypes, however, most C2 group patients did not have data available. Although data was unavailable for many patients, generally our subtyping system may potentially differ from current subtyping systems and provide additional value for patient diagnosis and prognosis. (Fig. 3B1) Our analysis also shows that the C1 subtype and the C2 subtype were significantly different in IDH1 mutations (Fig. 3B2), this accounts for the uneven distribution of MAPK subtypes within different glioma subtypes. IDH mutation could be at the origin of younger patient age^44^, and longer overall survival^45^. Thus, it is possible that the C2 group with longer overall survival, are younger patients. To explore if age was associated with the subtypes, we compared the ages of C1 and C2. Results showed that although C1 generally had an older average age than C2, there was a large variation with an overlapping age range from 20 to 75 years (Fig. 3B3). Hence, it is difficult to conclude that age accounts for the survival difference between the subtypes.

To investigate the potential role of the MAPK signaling pathway in the immunity of glioma, we analyzed the immune cell infiltration levels in glioma and compared the C1 subtype and the C2 subtype. The Xcell algorithms were used to estimate the immune cell infiltration levels. The results showed that the C1 and C2 subtypes of glioma had significant differences in multiple immune cells (Fig. 3C). In order to investigate whether these subtypes affected immune therapy, we compared the expression of immune checkpoints (CD274, TIGIT, CTLA4, LAG3, HAVCR2, PDCD1, SIGLEC15, and PDCD1LG2^46^) in the two subtypes. The results indicated that, compared to the C2 subtype, the C1 subtype had significantly higher expression of HAVCR2, CD274, PDCD1, CTLA4, SIGLEC15, PDCD1LG2, and LAG3, but significantly lower expression of TIGIT (Fig. 3D). To further investigate the impact of these subtypes on immune therapy, we compared the predicted responses to immune checkpoint blockade (ICB) in the two subtypes. Our analysis showed that the C1 subtype had a higher TIDE score than the C2 subtype (Fig. 3E bottom). Only 44.4% (72 out of 162) of C1 subtype glioma patients were predicted to respond to ICB treatment, while 57.9% (232 out of 401) of C2 subtype glioma patients were predicted to respond to ICB treatment (Fig. 3E top). These results suggested that C1 subtype glioma patients have more chance be sensitive to immunotherapy. As a result, we proposed that the MAPK signaling pathway signature can be used as a predictive factor for ICB therapy.

Moreover, it is valuable to cluster samples into more subtypes considering future application in clinical glioma treatment, thus we conducted a 4 subtype clustering for future reference (S-Fig. 2). We hope the clinical treatment of glioma can be benefited from the glioma subtype based on the glioma up-regulated MAPK signaling pathway gene set.

### The construction of a machine-learning risk model

**Figure 4:**
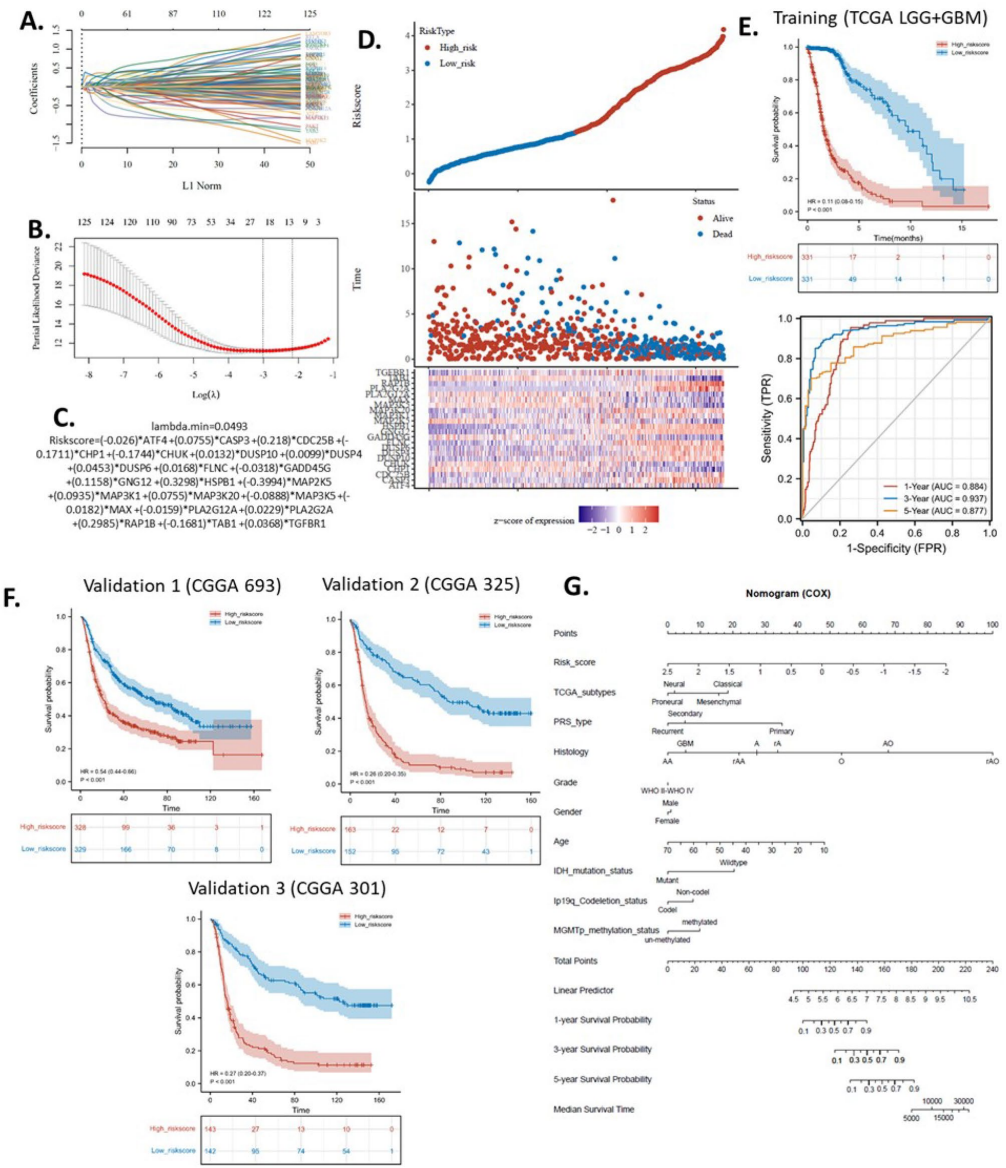
The machine-learning glioma risk model based on the up-regulated MAPK signaling pathway gene set. (**A**) Coefficients of genes shown by lambda parameter. (**B**) Partial likelihood deviance versus log (λ) drawn using the LASSO Cox regression model. (**C**) The algorithm of the LASSO Cox regression model. (**D**) Risky factor analysis of the risk model. (**E**) Overall survival KM plots with time-dependent ROC of the risk model of the training cohort (TCGA LGG + GBM). (**F**) Overall survival KM plots of three validation cohorts from CGGA. Data were normalized by the TPM method. (**G**) Prognostic nomogram of glioma patients using the risk model with other clinical factors. The “ggplot2” package was utilized to plot the figures with R software.

In machine learning, LASSO (least absolute shrinkage and selection operator) is a regression analysis method that performs both variable selection and regularization in order to enhance the prediction accuracy and interpretability of the resulting statistical model. In this study, we utilized LASSO regression to select the genes included and the coefficients in the prognostic model from the glioma-up-regulated MAPK signaling pathway gene set. TCGA LGG + GBM cohort was used to train the model. The best fit lambda (λ) was 23 (Fig. 4A,B). The algorithm of the risk model was shown in Fig. 4C, with 23 genes included with optimized coefficients.

To demonstrate the accuracy of the prediction, we performed a single risk assessment of the risk model by dividing the patients into a high-risk group and a low-risk group. The survival status of the patients was plotted based on their risk level. Overall, the results showed that in the high-risk group, death and survival points tended to be concentrated at lower survival times, while in the low-risk group, death and survival points were more dispersed over a wider range of survival times (Fig. 4D). We performed a Kaplan–Meier survival analysis to compare the survival rates of the two groups in the training cohort. The results showed that there was a significant difference between the groups in terms of survival. To assess the effectiveness of the risk model in predicting survival, we calculated the time-dependent receiver operating characteristic (ROC) curve. The results showed that the area under the curve (AUC) for predicting overall survival at 1, 3, and 5 years were 0.88, 0.93, and 0.87, respectively. (Fig. 4E). An AUC of over 0.9 is regarded as outstanding and an AUC of 0.8–0.9 is regarded as excellent. Thus the model was trained to be excellent or outstanding. Then, we validated the model using three independent cohorts from the CGGA database, including CGGA 693, CGGA 325, and CGGA 301. The KM plot and survival analysis revealed that the model performed well in all three external glioma cohorts (Fig. 4F). In addition, to develop a practical strategy for glioma prognosis using the risk model, we construct a nomogram of the risk model with other clinical factors (Fig. 4G).

### Cancer associations of the risk model

**Figure 5:**
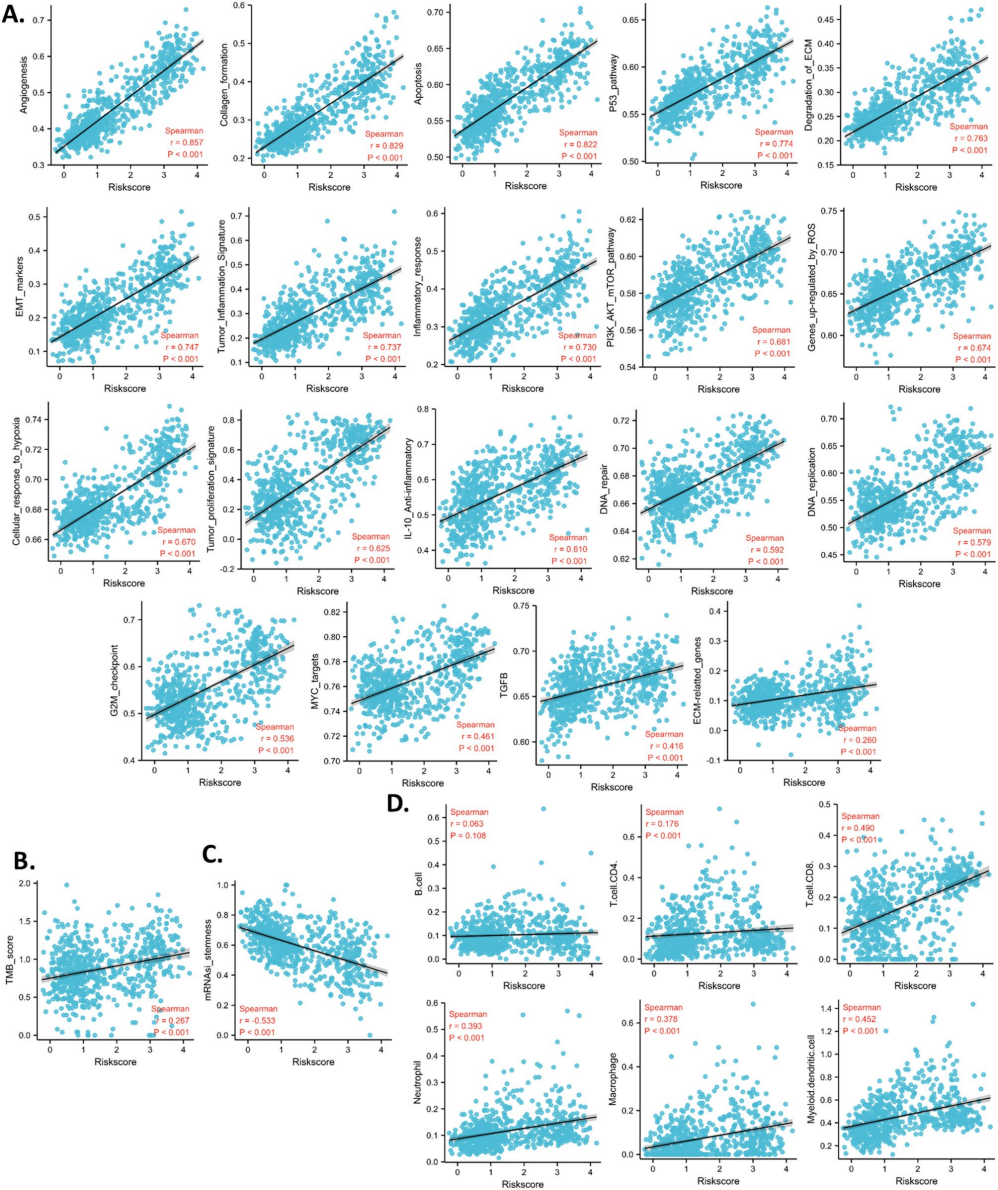
Association of the risk model and tumor scores. TCGA data were used to calculate all analyses in this figure. (**A**) Correlation between the risk model and pathway scores. (**B**) Correlation between the risk model and tumor mutation burden (TMB). (**C**) Correlation between the risk model and stemness. (**D**) Correlation between the risk model and immune cell infiltration levels. The “ggplot2” package was utilized to plot the figures with R software.

To further understand the associations between the risk model and pathways in glioma, we calculated 19 signaling pathway scores and analyzed the correlation between the risk score and these signaling pathway scores. Results revealed that the risk score was significantly positively correlated with all the signaling pathway scores. The coefficients of correlations of angiogenesis, collagen formation, and apoptosis were over 0.8. The coefficients of correlations of the p53 pathway, degradation of ECM, EMT markers, and tumor inflammation signature were between 0.7 to 0.8. While the coefficients of the correlation of the rest of the signatures were between 0.4 to 0.7, except for ECM-related genes (0.260) (Fig. 5A). These analyses suggested that the risk score was correlated to multiple cancer signals. In addition, the risk score was positively correlated with TMB (Fig. 5B). These analyses suggested that the risk score might be used to predict the mutation rate in glioma. In addition, the risk score was associated with the stemness of the glioma (Fig. 5C). Moreover, we calculated the correlation between the risk score and infiltration levels of the immune cells. Data revealed that the risk score was positively correlated with Tcell CD4, Tcell CD8, neutrophil, macrophage, and myeloid dendritic, but was not corrected with B cell. These analyses suggested that the risk model might be able to predict the glioma immune microenvironment (Fig. 5D).

### Protein–protein interaction network and hub genes identification

**Figure 6:**
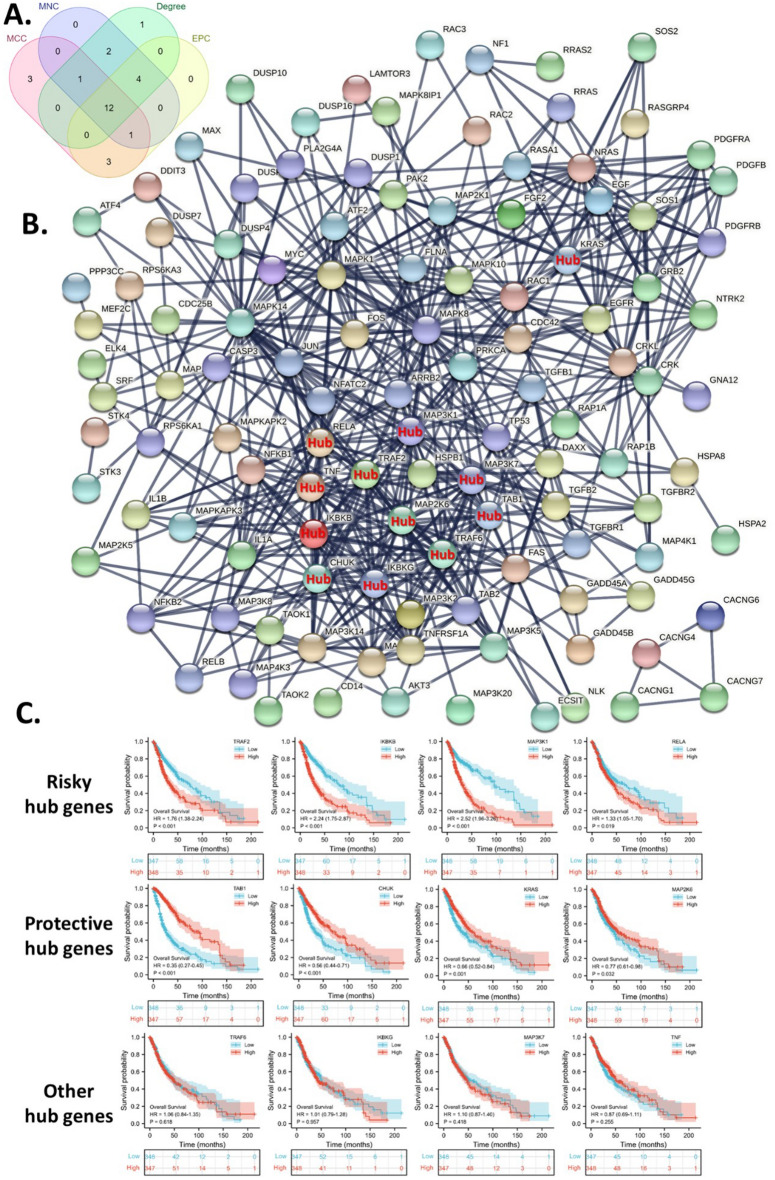
Identification of risky hub genes from the up-regulated MAPK signaling pathway gene set. (**A**) The intersection analysis of hub gene sets identified by four hub algorithms. (**B**) Protein–protein interaction network of the up-regulated MAPK signaling pathway gene set with common hub genes. (**C**) The survival associations of the common hub genes. The “ggplot2” package was utilized to plot the figures with R software.

To display the interconnection of survival-critical HP-upregulated genes, we constructed a protein–protein interaction network. In addition, we also identified the top 20 hub genes in the network using the four algorithms, including the “MCC”, “MNC”, “EPC”, and “degree”. Then we identified the common hub genes of the four calculations. Thus, we obtained 12 hub genes, including CHUK, IKBKB, IKBKG, KRAS, MAP2K6, MAP3K1, MAP3K7, RELA, TAB1, TNF, TRAF2, and TRAF6, which were displayed in the protein–protein interaction network (Fig. 6A,B). To further identify key hub genes for glioma patients, we analyzed the survival association of these hub genes. These analyses suggested that TRAF2, IKBKB, MAP3K1, and RELA were associated with worse survival. On the other hand, TAB1, CHUK, KRAS, and MAP2K6 were associated with better survival (Fig. 6C).

### Example drug prediction for a hub protein targets IKBKB

**Figure 7:**
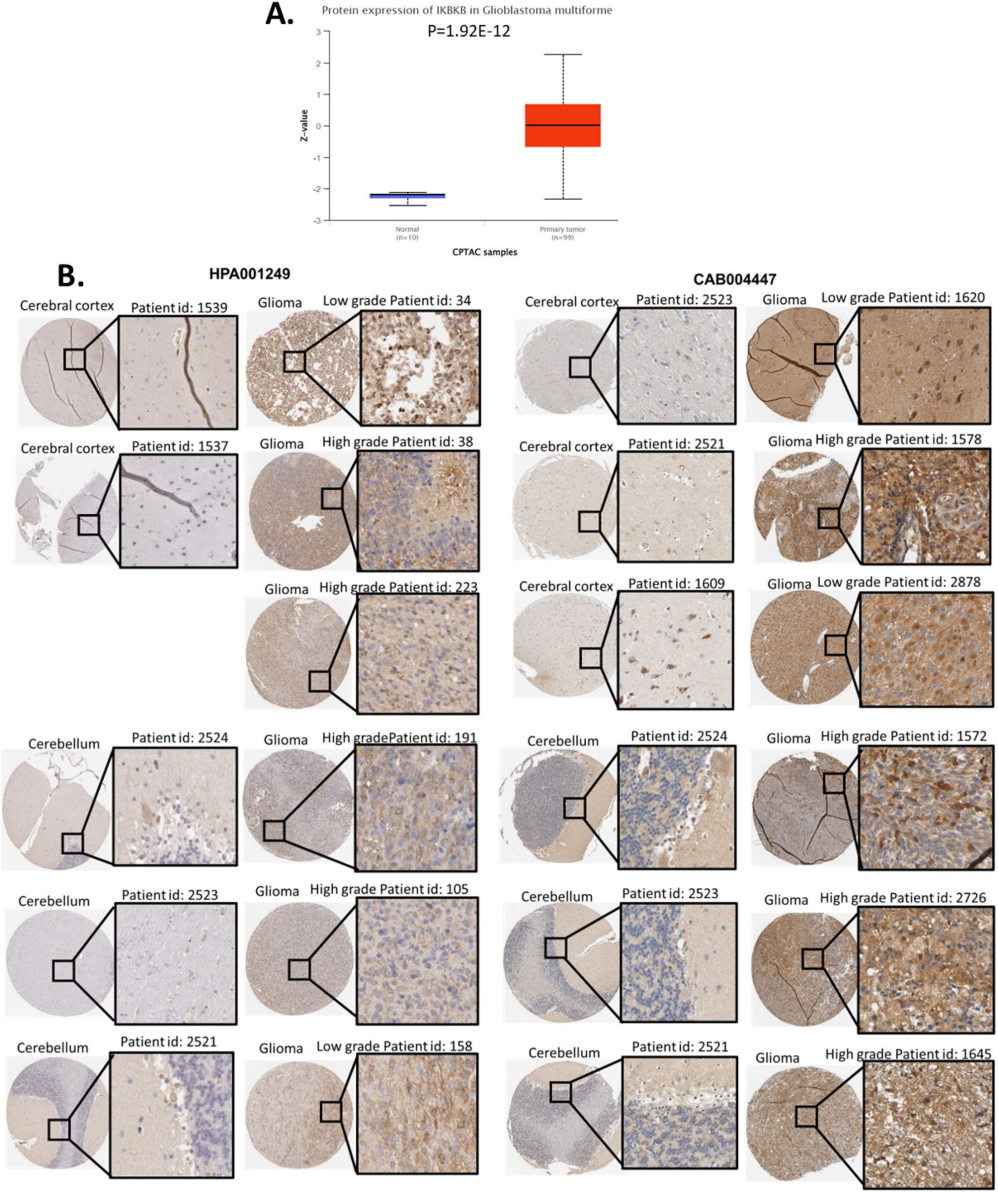
Protein expression of IKBKB, an example of a key biomarker in the MAPK pathway. (**A**) Expression of IKBKB proteins in glioma and normal tissues. (**B**) Representative protein staining images of IKBKB in glioma and normal brain tissues.

To demonstrate the application and potential value of this study in clinical glioma, we reported an example biomarker for protein overexpression in glioma, IKBKB. The CPTAC data suggested that IKBKB was overexpressed in GBM glioma compared with normal brain tissues (Fig. 7A). To further investigate the overexpression of IKBKB in glioma, we observed the stainings of IKBKB protein in glioma and brain tissues with two different antibodies. Both high-grade glioma and low-grade glioma were included. The HPA database does not provide the LGG/GBM category for these samples. The images strongly suggested that IKBKB protein expression was much higher than that in normal tissues (Fig. 7B).

### Separate clustering analysis for LGG and GBM

**Figure 8:**
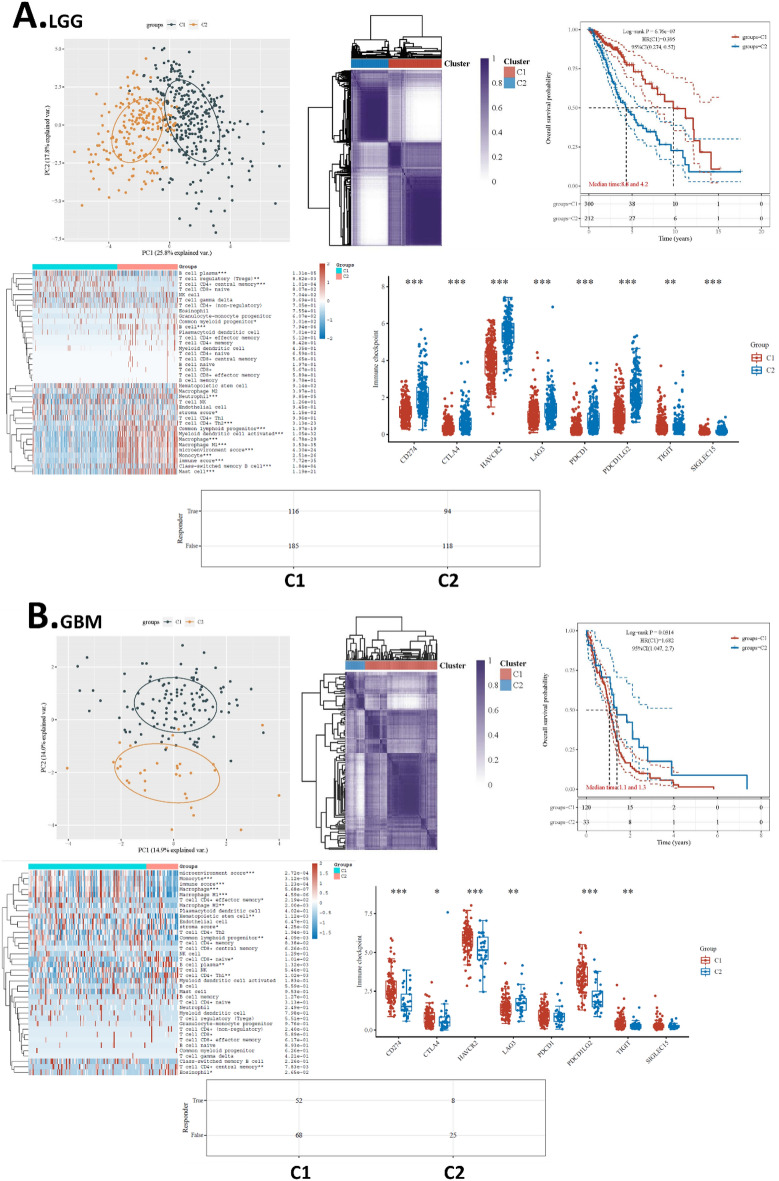
Separate clustering analysis for LGG and GBM. (**A**) Clustering analysis for LGG. (**B**) Clustering analysis for GBM. On the top of each panel from left to right are the PCA plot of the consensus clustering, Cluster trees and consensus matrix of subtypes, and overall survival KM plots of the subtypes respectively. On the bottom of each panel are the heat map of immune cell infiltration of the subtypes, immune check-point levels of the subtypes, and predicted ICB response of the subtypes respectively.

Given that glioma subtypes are distinct, a major concern in the analysis is raised because of the combination of low- and high-grade gliomas. Hence, we conducted separate clustering analyses for LGG and GBM respectively, and analyzed their survival and immunity association. The clustering of subtypes was conducted in the same way we have done for the overall glioma data set. The LGG and GBM samples were clustered into two clusters respectively. Results showed that, for LGG, MAPK-based clustering subtypes were significantly different in overall survival. The subtypes were also different in many immune cell infiltration levels and the levels of all immune checkpoints. The prediction suggested that for LGG, 38.5% (116/301) of patients responded to immune therapy, while 44.3% (94/212) of patients responded to immune therapy. (Fig. 8A) On the other hand, for GBM, MAPK-based clustering subtypes were also significantly different in overall survival. The subtypes were different in many immune cell infiltration levels and 6 of the 8 immune checkpoints. The prediction suggested that for GBM, 43.3% (52/120) of patients responded to immune therapy, while 24.2% (8/33) of patients responded to immune therapy. (Fig. 8B) Therefore, these data supported that MAPK is critical not only for overall glioma but also for LGG and GBM respectively.

## Discussions

The MAPK signaling pathway has been linked to the development of glioma and was reportedly activated in this process^9,10^. Yet, these conclusions were based on determining and observing several key proteins in the pathway. In our project, we comprehensively analyzed the expression alterations of all genes in the MAPK signaling pathway. Our results supported the previous assumption that this pathway affects glioma because, among 267 genes, 127 genes were up-regulated in glioma with only 8 genes down-regulated. The large proportion of genes overexpressed in glioma indicated that this pathway was activated and might be a unique target for glioma cells but not normal brain cells. Furthermore, the overexpression of these gene sets also suggested a diagnostic value of this pathway. Although a number of papers reported a single gene signature for diagnosis^47–50^, using a pathway signature of a set of genes for diagnosis can be more stable than using a single biomarker gene.

As half of the MAPK signaling pathway genes were highly expressed in the glioma over the normal brain, detecting these genes in the glioma might be easier because the detection had a lower background expression from normal brain cells. Therefore, we were interested in developing these overexpressed genes for glioma prognosis. However, the value of this pathway genes for prognosis was not able to be evaluated as a single gene signature because the pathway was high-dimensional data. To address this issue, we utilized cluster analysis to decrease the dimension of the data and thereby investigated the impact of this pathway on glioma. Cluster analysis has been wildly used in the field^51^. Our cluster provided a view to observe the association between the MAPK signaling pathway and glioma survival. Results suggest that the clustering efficiently distinguish patients with different survival rate. One of the subtypes had significantly poorer survival compared to the other subtype. Notably, the difference in progression-free survival indicated that this pathway might affect the treatment of glioma. Progression-free survival represents the time from treatment initiation until disease progression, which is a direct indicator of the clinical benefit of drugs or immune therapy response^52^. Hence, we suggested that the MAPK signaling pathway might make a difference during the treatment of glioma.

Other analyses also revealed that the MAPK signaling pathway might have impacts on cancer stemness, mutation burden, and immune microenvironment. MAPK phosphorylation has been suggested to regulate the immune response^53^. So far, the direct involvement of this pathway in immunity was least reported, yet, a few studies have revealed potential indirect effects of it on immune cells. For example, this pathway was reported to play a role in immune escape under PDL1 immune therapy^54^. These results were generally consistent with our assumption that this pathway can affect the response of the immune therapy, just as we demonstrated in the analysis of the predicted immune checkpoint blockade response.

A previous study reported TUC338 as a prognostic factor for tumors whose effects were medicated by the MAPK signaling pathway^55^. Similarly, in colon cancer, a prognosis-associated gene HNRNPA2B1 was reported to affect cancer cells through the MAPK signaling pathway^56^. However, so far, this study is the first study that developed the MAPK signaling pathway prognostic model. To maximize the prognostic value of the MAPK signaling pathway genes, we included all the glioma up-regulated MAPK signaling pathway genes and constructed risk models using the LASSO method. LASSO is a machine learning method that combines variable selection and regularization in order to improve the accuracy and interpretability of regression models^57^. The training cohort had 663 cases which enable sufficient training for the model. The model was further validated with three external cohorts with relatively large case numbers. CGGA 693, CGGA 325, and CGGA 301 had 693, 325, and 301 cases respectively, thus, we believe the model was reliable. In addition, the model was also found to correlate with many other pathway scores, indicating that the model might have multiple applications.

The overexpression of the MAPK signaling pathway provided three advantages: (1) easy detection with low background, (2) distinguishing markers between glioma and normal brain cells, and (3) potential specific drug targets for glioma. Previous studies have proposed and demonstrated several drugs that targeted the MAPK signaling pathway in glioma cells^16–18^. However, non of these studies applied a systematic screening method to discover potential drugs and key targets for the MAPK signaling pathway in glioma. Recently developed bioinformatic methodologies and databases enable better strategies to screen and predict the potential drugs and key targets in this pathway in glioma. In this study, with bioinformatics data, we narrowed the key target to a most promising protein: IKBKB. The inhibitor of nuclear factor-kappa B kinase subunit beta (IKBKB) works by breaking down the inhibitor of kappa B, which allows nuclear factor-kappa B to be activated and carry out its functions. This process helps to prevent the inhibition of nuclear factor-kappa B^58^. The expression of IKBKB has been reported to regulate some cancer types, such as breast cancer^59^, kidney cancer^60^, and lung cancer^61^. In glioma, IKBKB has been found to regulate apoptosis^62^, invasion^63^, and migration^63^. These results support our hypothesis that IKBKB might be a drug target for glioma treatment. In fact, previously, IKBKB has been suggested as a potential target for cancer treatment^64^, but few studies have been screening and discovering the potential drugs for this pharmacological target. We hoped our screening and prediction provided potential drugs and targets for future studies of the MAPK signaling pathway in glioma.

Granted, this study was subjected to a limitation: this study is based on open data without experimental validation, thus the mechanistic proofs are weak. Nevertheless, we hope the finding that the MAPK signaling pathway was overexpressed in glioma with prognostic value can provide novel insight into glioma treatments, and the example drug prediction of the key hub protein IKBKB provided a strategy for future drug discovery.

### Supplementary Information

Supplementary Information.

## Data Availability

The datasets used in this study are all publicly available and the source has been mentioned in the method section. The raw data used in this study were sourced from the paper, and the raw analysis data can be obtained by contacting the corresponding author upon request.
