# Supplementary material for: MAPK signaling pathway-based glioma subtypes, machine-learning risk model, and key hub proteins identification
Source: Sci Rep. 2023 Nov 4;13:19055. doi: 10.1038/s41598-023-45774-0 (PMC10625624; doi:10.1038/s41598-023-45774-0)
Supplement: Supplementary file 1 — Supplementary Information. [file 41598_2023_45774_MOESM1_ESM.docx]

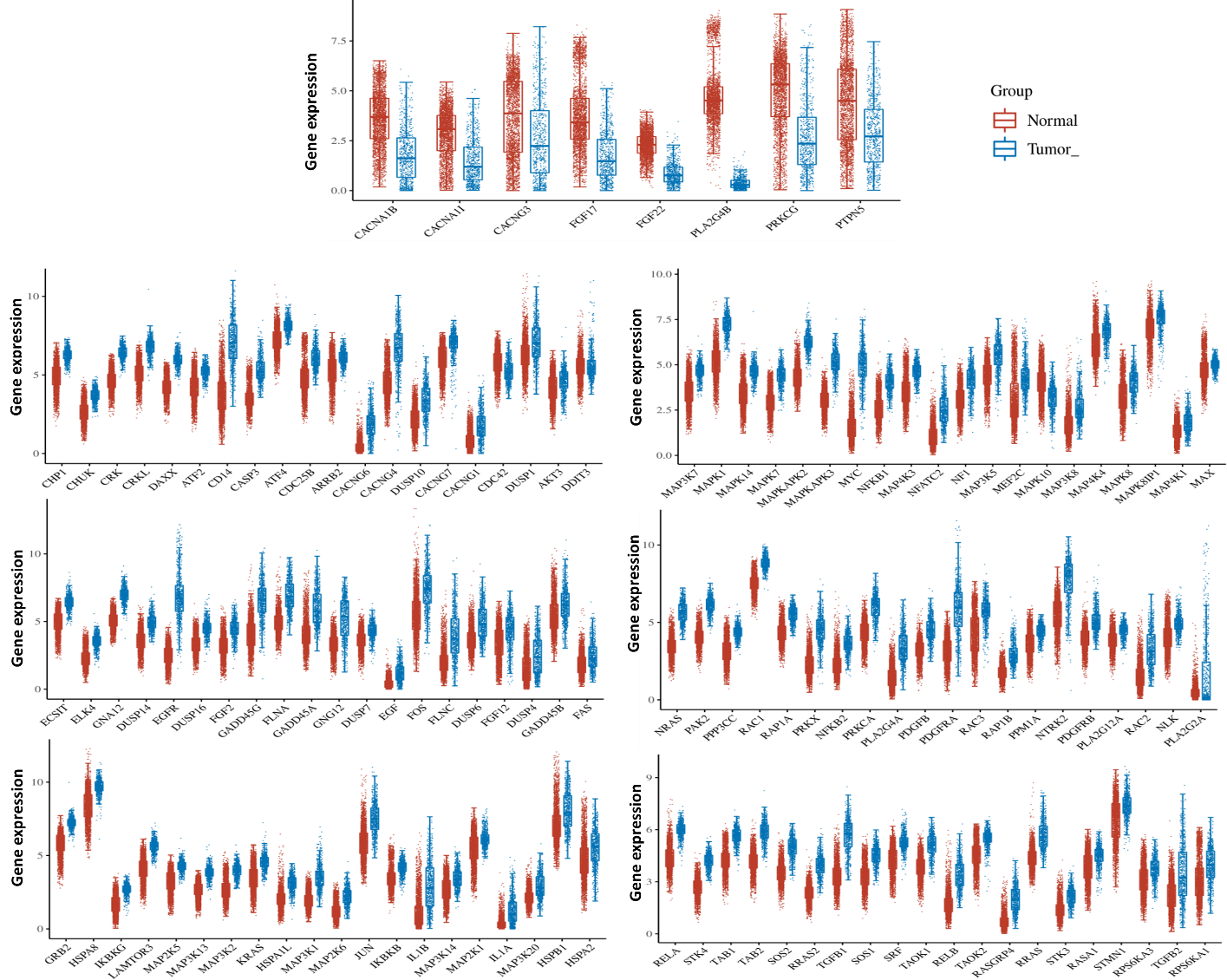


**S-Figure 1.** Expression of differentially expressed MAPK signaling pathway genes in glioma. The “ggplot2” package was utilized to plot the figures with R software.


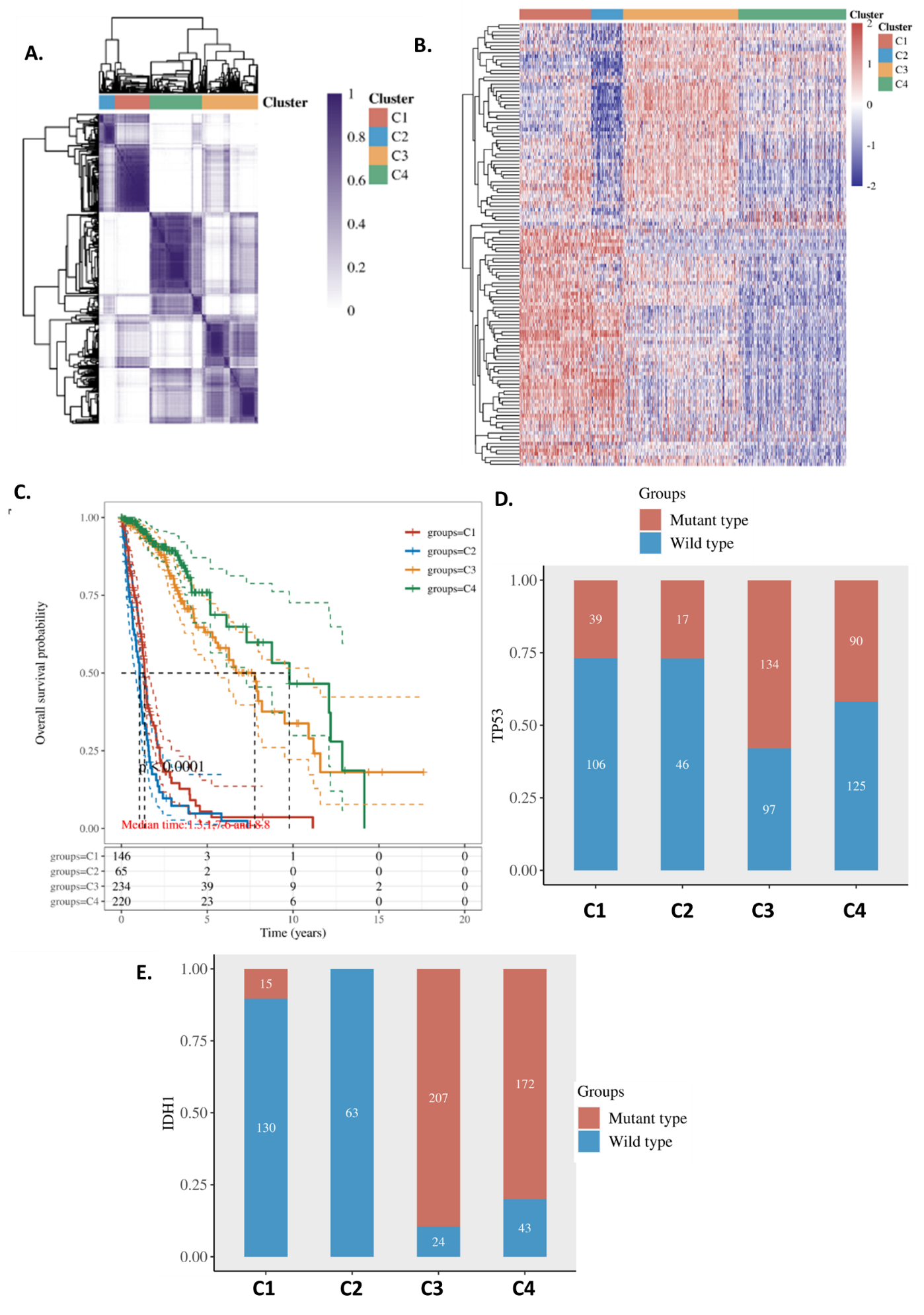


**S-Figure 2.** Glioma subtype based on the glioma up-regulated MAPK signaling pathway gene set (Clustering to 4 subtypes). **A.** Consensus matrix and cluster trees of subtypes. **B.** expression heatmap of subtypes. **C.** Overall survival of subtypes. **D.** Subtype difference in TP53 mutation. **E.** Subtype difference in IDH1 mutation. The “ggplot2” package and “pheatmap” package were utilized to plot the figures with R software.
